# Preparation of Carbon Nanotubes/Alumina Hybrid-Filled Phenolic Composite with Enhanced Wear Resistance

**DOI:** 10.3390/ma16072772

**Published:** 2023-03-30

**Authors:** Siti Shuhadah Md Saleh, Mohd Firdaus Omar, Hazizan Md Akil, Muhammad Helmi Abdul Kudus, Mohd Mustafa Al Bakri Abdullah, Andrei Victor Sandu, Petrica Vizureanu, Khairul Anwar Abdul Halim, Mohamad Syahmie Mohamad Rasidi, Syarifah Nuraqmar Syed Mahamud, Ion Sandu, Norlin Nosbi

**Affiliations:** 1 Faculty of Chemical Engineering & Technology, Universiti Malaysia Perlis (UniMAP), Kangar 01000, Perlis, Malaysia; 2 Centre of Excellence Geopolymer and Green Technology (CEGeoGTech), Universiti Malaysia Perlis, Kangar 01000, Perlis, Malaysia; 3 School of Materials and Mineral Resources Engineering, Engineering Campus, Universiti Sains Malaysia, Nibong Tebal 14300, Pulau Pinang, Malaysia; 4 School of Mechanical Engineering, Engineering Campus, Universiti Sains Malaysia, Nibong Tebal 14300, Pulau Pinang, Malaysia; 5 Faculty of Material Science and Engineering, Gheorghe Asachi Technical University of Iasi, 41 D. Mangeron St., 700050 Iasi, Romania; 6 Romanian Inventors Forum, Str. Sf. P. Movila 3, 700089 Iasi, Romania; 7 National Institute for Research and Development for Environmental Protection INCDPM, 294 Splaiul Independentei, 060031 Bucharest, Romania; 8 Technical Sciences Academy of Romania, Dacia Blvd 26, 030167 Bucharest, Romania; 9 Arheoinvest Platform, Alexandru Ioan Cuza University of Iasi, Bd. Carol I, No. 22, Iasi 700506, Romania; 10 Academy of Romanian Scientists AOSR, 54 Splaiul Independentei St., Sect 5, 050094 Bucharest, Romania; 11 Department of Mechanical Engineering, Centre for Corrosion Research (CCR), Institute of Contaminant Management for Oil and Gas (ICM), Universiti Teknologi PETRONAS, Seri Iskandar 32610, Perak, Malaysia

**Keywords:** hybrid, polymer composite, carbon nanotubes (CNTs), wear, chemical vapour deposition

## Abstract

Hybrid fillers can be produced via various methods, such as physical mixing and chemical modification. However, there is a limited number of studies on the effect of hybridisation on the mechanical performance of hybrid filler-reinforced polymer composites, especially in the context of wear performance. This study investigated the wear resistance of carbon nanotubes (CNTs)/alumina hybrid-filled phenolic composite, where two hybrid methods were used to produce the CNTs/alumina hybrid filler. The CNTs/alumina (CVD hybrid) was synthesised using the chemical vapour deposition (CVD) method, whereas the CNTs-/alumina (physically hybrid) was prepared using the ball milling method. The CNTs/alumina hybrid filler was then used as a filler in the phenolic composites. The composites were prepared using a hot mounting press and then subjected to a dry sliding wear test using a pin-on-disc (POD) tester. The results show that the composite filled with the CVD hybrid filler (HYB composite) had better wear resistance than the composite filled with physically hybrid filler (PHY composite) and pure phenolic. At 5 wt%, the HYB composite showed a 74.68% reduction in wear, while the PHY composite showed a 56.44% reduction in wear compared to pure phenolic. The HYB composite exhibited the lowest average coefficient of friction (COF) compared to the PHY composite and pure phenolic. The average COF decreased with increasing sliding speeds and applied loads. The phenolic composites’ wear and average COF are in the order HYB composite < PHY composite < pure phenolic under all sliding speeds and applied loads.

## 1. Introduction

Polymers are extensively used in engineering components due to their self-lubricating properties. Many polymer-based composites have been used for sliding contact against polymers, metals, and other materials [1,2]; however, many problems caused by friction and wear (tribological properties) have been reported when contact is involved. To overcome these problems, the polymers were reinforced with other materials to improve their tribological properties, making them more durable for wear-related applications [3,4,5,6,7].

Phenolic is a thermoset polymer that is widely used in polymer composite systems. Phenolic is ubiquitous in electrical applications, automobiles, aerospace, military weapons, and sports goods due to its superior mechanical strength, heat resistance, dimensional stability, and high chemical, acid, and water resistance [8]. Phenolic is also one of the essential ingredients used in friction material as it is a strong binder, and high-strength binders improve the overall friction materials’ performance [9]. There is literature that discusses the improvement of phenolic’s properties when reinforced with fillers such as alumina, calcium carbonate, silicon carbide, talc, copper, carbon black, graphite, and CNTs [10,11,12]. 

The use of a single filler in polymer matrix composites (PMCs) can no longer meet the demands of advanced PMC applications, which require multifunctional properties from a combination of two or more fillers and a hybrid system [13,14,15]. Attempts to combine several fillers or hybrid materials to form composites are not recent endeavours. For example, clay, iron, mica, and carbon were used as fillers in automotive brake pads [16]. In these applications, more than one filler component is usually used (hybrid). Hybrid systems attract much attention due to their multifunctional properties, which are superior to those of a single constituent system. Hybridisation is achieved by mixing two or more different materials [17,18]. Carbon nanotubes (CNTs) have been used as reinforcements for polymer composites due to their superior thermal conductivity, mechanical properties, and outstanding tribological properties [19,20,21]. CNTs improve mechanical properties when added to a metallic [22,23,24,25], polymeric, [26,27] or ceramic matrix [28,29]. In some works, CNTs enhanced wear resistance in polymer composites [30,31,32,33,34,35,36]. Hwang et al. reported that CNTs improved the friction materials’ wear resistance on the brake friction materials’ tribological performance [36]. 

Multi-walled carbon nanotubes (MWCNTs) reported several advantages over single-walled carbon nanotubes (SWCNTs) in the context of mechanical properties. MWCNTs have larger diameters and thicker walls, which makes them more resistant to deformation and buckling under external forces [37,38]. This means that MWCNTs are generally stronger and more rigid than SWCNTs. MWCNTs also have a unique structure that allows for interlayer sliding between adjacent walls, providing an additional mechanism for energy dissipation and enhancement of mechanical properties [39,40]. MWCNTs can absorb more energy before failure than SWCNTs. These factors make MWCNTs more suitable for applications requiring high strength and stiffness, such as developing advanced composite materials and improving wear resistance. Due to the above reasons, MWCNTs were selected for synthesis and reinforcement with the phenolic matrix in this work.

The main problem with using CNT-reinforced composites is the agglomeration of CNTs, which prevents the matrix resin from penetrating the CNT. Due to its excellent properties, alumina (Al_2_O_3_) is hybridised with CNTs [41]. The hybrids can be prepared by methods such as simple sonication, milling, and chemical vapour deposition (CVD) methods [42,43]. The sonication and milling methods (physically hybrid) could potentially damage the CNTs’ structure [44]; the former is not viable for mass production, while the latter will cause the CNTs not to merge with the alumina’s surface, resulting in an inhomogeneous dispersion and the formation of CNT clusters within the polymer composite [41]. 

In order to reinforce the composite system, the hybrid materials, i.e., the CNTs/alumina, need to be physically and chemically attached. Hybridising the micrometre-sized alumina and nanometre-scaled CNTs (CNTs attached on alumina’s surfaces) would result in the cluster effect as the vacancies left by the micrometre-sized particles can be filled by the nanometre-sized CNTs [45]. The hybridisation of CNTs/alumina using the chemical vapour deposition (CVD) method is recommended to enhance dispersion without damaging the structure while preserving the properties of the CNTs. Hybridisation via CVD is expected to result in more homogenous compounds that can be used as fillers in composites.

In this study, the hybridisation of the CNTs/alumina was carried out using the CVD and milling methods. Limited data are available from the literature for the tribological properties of CNTs/alumina hybrid synthesised using CVD, and thus, the goal of this study is to investigate the tribological properties of CNTs/alumina (CVD hybrid) and CNTs/alumina (physically hybrid)filled phenolic composites. Also, the wear and the coefficient of friction of the phenolic composites were tested to determine their effect on the tribological properties of the CVD hybrid filler/phenolic and physically hybrid filler/phenolic composites.

## 2. Materials and Method

### 2.1. Preparation of Hybrid Filler

The CNTs/alumina (CVD hybrid) was synthesised using the chemical vapour deposition (CVD) method. The co-precipitate method was used to prepare the Ni/alumina catalyst. Catalyst salts and Nickel (II) nitrate hexahydrate (Ni(NO_3_)_2_·6H_2_O) (0.01 mol, 98% purity) (Merck Chemicals, Darmstadt, Germany) were mixed with Al powder (0.38 mol, 99% purity) (Euro Chemo-Pharma Sdn. Bhd., Pulau Pinang, Malaysia)) in distilled water. Sodium hydroxide (NaOH) (0.02 mol 98% purity) (Merck Chemicals, Darmstadt, Germany) was dissolved in distilled water (50 mL) and added into the previous mixture while being constantly stirred. The mixture was precipitated for 24 h. The precipitate was washed, filtered, and dried at 110 °C for 2 h, then calcined at 900 °C. Next, the catalyst was reduced in hydrogen at 400 °C for 2 h. To produce the CNTs, the reduced catalyst was reacted with a methane/nitrogen mixture at a ratio of 1:7 at 800 °C for 30 min. The production of CNTs was carried out in a custom-made CVD horizontal tube furnace equipped with a precise gas flow control unit. The physical milling method produced the CNTs/alumina (physically hybrid). CNTs with 95% purity (MWCNTs, purchased from SkySpring Nanomaterials Inc., Houston, USA) and alumina (Sigma Aldrich, Germany) were mixed at a ratio of 23:77 using a ball milling machine for 48 h at 20 rpm, with ceramic ball media with diameters of 40–100 mm. The ratio of CNTs and alumina was calculated quantitatively based on an energy-dispersive X-ray (EDX) analysis of the CNTs/alumina (CVD hybrid) compound. Table 1 shows the EDX result for the CNTs/alumina hybrid synthesis using CVD method.

### 2.2. Preparation of Composite

The phenolic powder used in this study was procured from Pace Technologies. The CNTs/alumina hybrids were mixed with the phenolic powder using a ball milling machine for 24 h at 20 rpm. The ceramic balls used for milling had diameters of 40–100 mm. The mixtures of the CVD hybrid/phenolic and the physically hybrid/phenolic were pressed using a hot mounting press at 150 °C under 10 MPa of pressure for 10 min (5 min heating and 5 min cooling). Table 2 details the samples used in this study.

### 2.3. Wear and Friction Test

The wear and friction tests were performed using a pin-on-disc tester, per the ASTM G-99–05(2010). After being hot-mounted, the sample was cut into squares measuring 9 × 9 × 30 mm. The sample abrasion area measured 9 × 9 mm. The disc was made of stainless steel, ~10 mm thick and 188 mm in diameter. The disc surface was covered with abrasive paper made of silicon carbide (SiC) at grit 400 (Ø 20 mm). The sample was clamped onto a holder. The distance between the sample and abrasive paper, d, was 21 mm. The samples were run in dry sliding conditions and abraded against the abrasive paper to simulate abrasive wear conditions. The tests were run for 5 min for different loads and sliding speeds (applied loads of 10, 20 and 30 N, and sliding speeds of 0.033, 0.368 and 1.022 m/s). After the wear test, the weight lost from each sample was measured and recorded. The wear of each sample after each wear test was measured using a digital electronic balance with ±1 mg accuracy. The lowest weight loss would directly translate into the highest wear resistance.

### 2.4. Field Emission Scanning Electron Microscopy (FESEM)

A Field Emission Scanning Electron Microscope (LEO SUPRA 35VP, Carl Zeiss, Germany) was used to image the morphologies of the hybrid compound and the worn surface of the composites. Before imaging, the samples were coated with gold/palladium (Au-Pd) using a vacuum sputter chamber.

### 2.5. High-Resolution Transmission Electron Microscopy (HRTEM)

To further analyse the morphology of the hybrid compound and its composites, high-resolution transmission electron microscopy (Philip TECHNAI 20 (200 kV)) was used. The synthesised compound was sonicated in a 2% surfactant solution for 30 min. Then several drops of the solution were dripped into the holes in the carbon grids. The composite (HYB and PHY) samples were also characterised using HRTEM to image and analyse the fillers’ dispersion states. Samples 50 nm thick were prepared by cryo-ultramicrotomy using a microtome by Leica (Model Reichert-Jung Ultracut E). 

### 2.6. Rockwell Microhardness

Hardness testing was performed using a Rockwell microhardness testing machine (HRB100), and a 1/16 ball indenter was used to determine the hardness, per the ASTM D785.

## 3. Results and Discussions

### 3.1. Characterization of Physically Hybrid and CVD Hybrid Fillers

**Figure 1:**
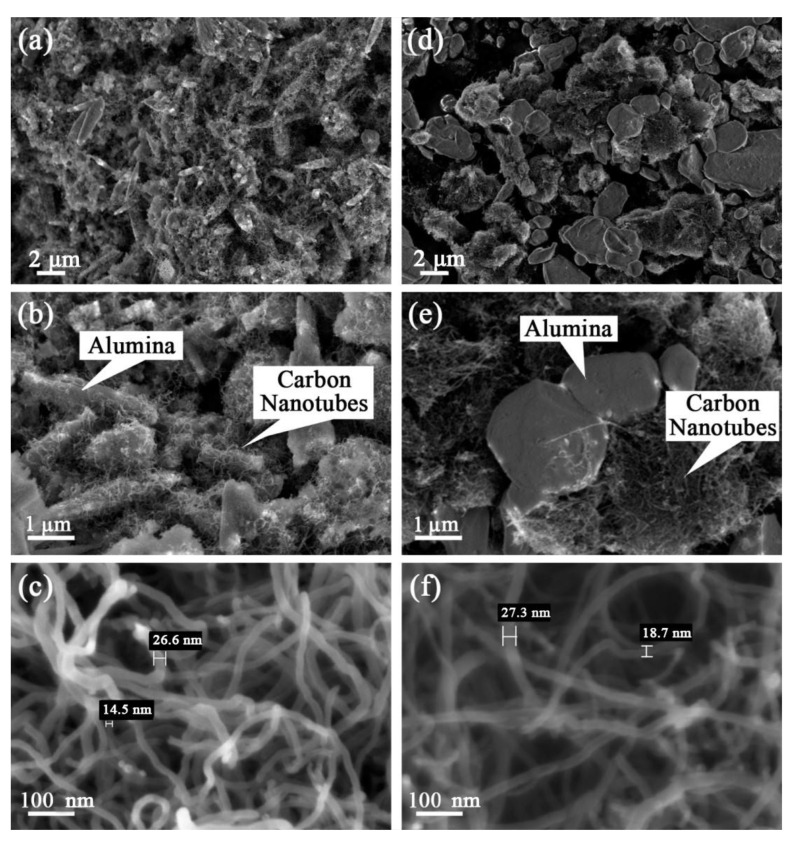
FESEM micrograph of the CNTs/alumina hybrid compound: (**a**–**c**) CVD hybrid and (**d**–**f**) physically hybrid.

**Figure 2:**
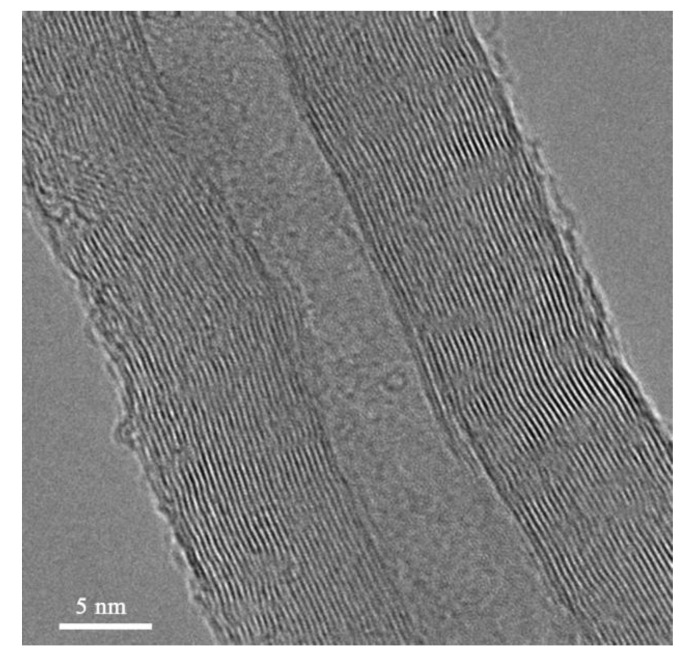
HRTEM image of CNTs/alumina CVD hybrid compound.

In order to confirm the distribution of the CNTs and alumina particles and further differentiate between the CVD hybrid and physically hybrid particles, a series of FESEM and HRTEM analyses were conducted. Figure 1a–c shows the FESEM micrograph of the CVD hybrid compound. It can be seen that in the CVD hybrid compound, the CNTs are securely attached to the alumina particle. The CNTs’ distribution on the alumina particles’ surface seems uniform and well-distributed. Figure 1d–f shows the FESEM of the CNTs/alumina (physically hybrid) compound, and it can be seen that the alumina and CNTs are not attached. It is also noticeable that the CNTs are not homogenously distributed on the alumina particles, and CNT agglomeration is evident, attributable to van der Waals interaction. Under HRTEM, the CVD hybrid CNTs were determined to be MWCNTs due to the presence of a multilayer wall and hollow structure, per Figure 2.

### 3.2. Effect of Hybrid Filler Loading on Wear Properties

**Figure 3:**
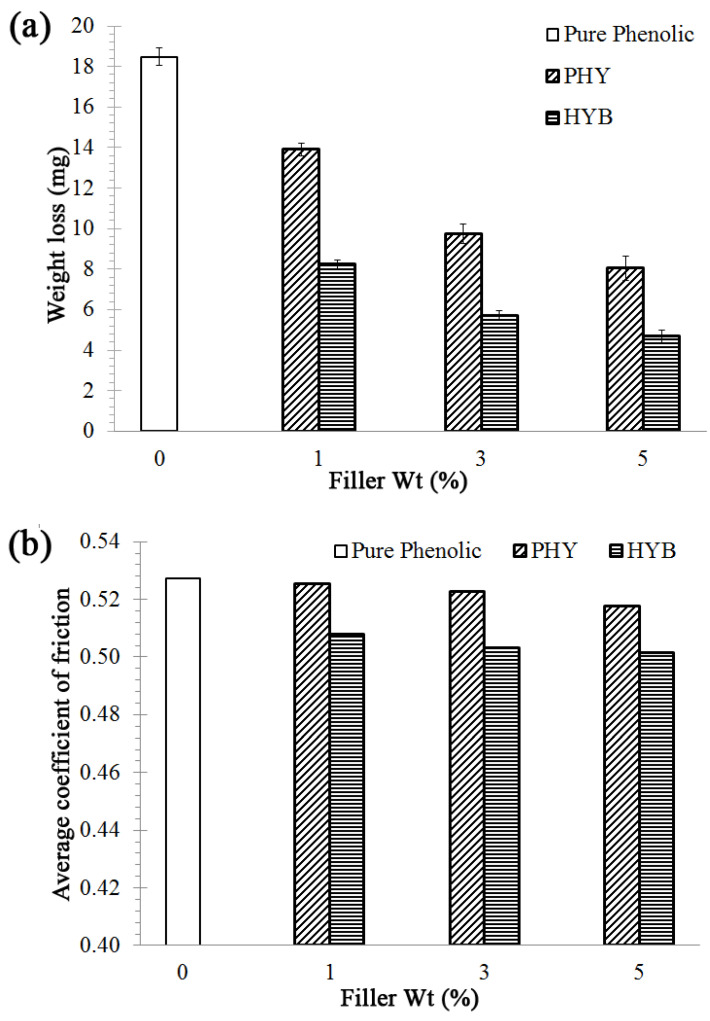
Properties of the HYB composite and PHY composite as a function of filler loading at 0.033 m/s sliding speed and 10 N applied load: (**a**) weight loss; (**b**) average coefficient of friction.

In order to elucidate the wear behaviour of phenolic-based composites filled with the CVD hybrid and physically hybrid fillers, the composites were analysed in terms of their wear (weight loss) and coefficient of friction (COF). Figure 3 shows the weight loss and the average COF of the HYB and PHY composites as a function of filler loading, using a 10 N applied load and 0.033 m/s sliding speed. Figure 3 shows the decrease in wear (weight loss) and decrease in average friction coefficient with increasing filler loading. The hybrid system composite (filled composite) demonstrated lower weight loss and average friction coefficient relative to pure phenolic. The hybrid filler affected the friction and wear behaviour of the composites. Ren G et al. found that when using graphene and PS-graphene as fillers in phenolic composites, filled phenolic composites demonstrated a much lower friction coefficient than the unfilled phenolic composite [46]. Liu and co-workers also reported that the filled MWCNT in ultra-high molecular weight polyethylene (UHMWPE) composite exhibited high wear resistance and low friction coefficient compared to pure UHMWPE [47]. According to Yan and Xue, incorporating carbon nanotubes into a polymer matrix improves the composite’s tensile and tribological properties [48]. An enhancement in wear resistance is evident after adding the hybrid fillers (HYB and PHY composite). Hybrid particles in the polymer matrix can resist the sliding shear stress on the contact surface and protect the polymer from being quickly torn away from the bulk body [49]. The 5 wt% HYB composites demonstrated better wear resistance and lower average COF than the 5 wt% PHY composites, which confirms that the addition of the CNTs/alumina (CVD hybrid) resulted in superior wear resistance to the phenolic-based composites. 

Generally, the HYB composites showed a lower weight loss (higher wear resistance) than the PHY composites and pure phenolic. In the case of the 5 wt% filler loading, the HYB composite showed a minimum weight loss of 4.68 mg and a 74.68% reduction in weight loss compared to pure phenolic. Meanwhile, the PHY composite’s weight loss was 8.05 mg, translating into a 56.44% reduction in weight loss compared to pure phenolic. The results confirmed that the HYB composite has better wear resistance than the PHY composite.

**Figure 4:**
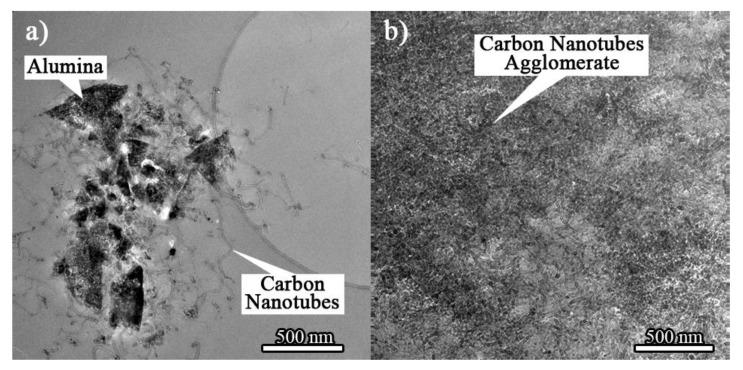
HRTEM image of (**a**) HYB composite; (**b**) PHY composite.

Figure 4a shows the HRTEM image of the HYB composite. It can be seen that the CNTs are attached to the alumina element and not agglomerated. This is because the CNTs are well dispersed in the matrix, and the alumina particles produce CNT networks in their place of agglomeration, which minimises the composites’ weight loss.

The improved dispersibility helps minimise the friction coefficient and enhances the anti-wear property of the composite [46]. In addition, the CNT networks formed by CVD can endure sliding shear stress on the contact surface and protect the polymer from being detached from the bulk body, which improves the composites’ wear resistance. 

The COF average of the HYB composite decreased with increasing filler loading, confirming that hybrid fillers can be self-lubricating. The hybrid filler strengthens the phenolic structure and decreases its adhesive and ploughing wear, resulting in significantly improved wear resistance. Figure 4b shows the HRTEM image of the PHY composite, where agglomeration of CNTs in the composite is evident. The CNTs are not distributed well on the alumina’s surface and are seen to be independent of the dispersion of the alumina particles. The filler cluster would cause the abrasive to wear and the filler to detach from the matrix, resulting in lower wear properties.

### 3.3. Effect of Sliding Speed on Wear and Coefficient of Friction

**Figure 5:**
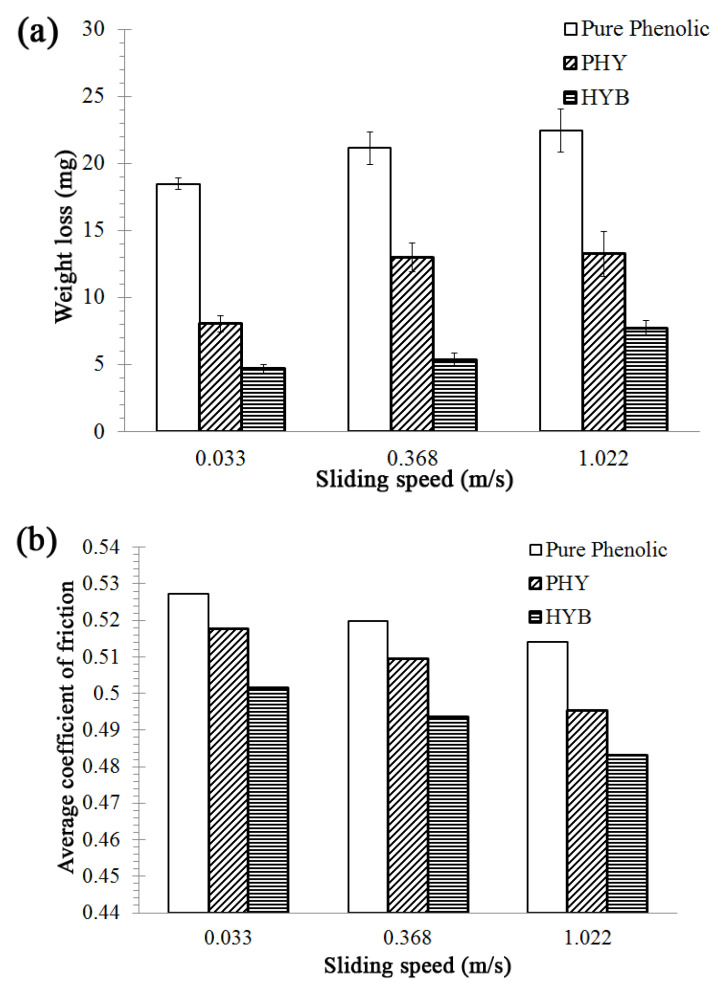
Properties of the HYB composite and PHY composite after wear tests for different materials with the variation in sliding speed (load: 10 N, Filler: 5 wt%): (**a**) weight loss, and (**b**) average coefficient of friction.

Figure 5 shows the weight loss and average COF of the HYB and PHY composite with 5 wt% filler loading with 10 N applied load under three different sliding speeds (0.033, 0.368, and 1.022 m/s). Figure 5 shows the increase in wear (weight loss) with increasing sliding speed for all composites. The weight loss of the pure phenolic is higher than that of the PHY and HYB composites. In the case of the filled composites, the wear (weight loss) slightly increased, while in the case of the HYB composites, the weight losses were 4.680 mg, 5.33 mg, and 7.71 mg, corresponding to sliding speeds of 0.330 m/s, 0.0368 m/s and 1.022 m/s, respectively. For the PHY composites, the weight losses were 8.05 mg, 12.99 mg, and 13.24 mg, respectively.

It can be seen in Figure 5 that the average COF of the composites decreases with increasing sliding speed. The HYB composite shows the lowest average COF, slightly decreasing with increasing sliding speed. The composites’ weight loss and average COF are in the order of HYB composite < PHY composite < pure phenolic at all sliding speeds. The friction phenomenon is susceptible to changes in the sliding conditions. The mechanism of wear involves the formation of debris particles [50,51]. The sliding action during the wear test would create shear stress on the phenolic interface surface and form a thin layer of polymer debris at the interface. This debris is then positioned on the counter face of the abrasive paper, which minimises frictional contact. The presence of this transfer film between the sliding surfaces significantly affects the friction and wear behaviour [50]. With increasing sliding speed, wear debris can form on the contact surface, which decreases COF. As a result, the wear (weight loss) increases with increasing sliding speed. It should also be mentioned that the composites’ interfacial temperature also affects wear properties [52]. 

Long periods of sliding at higher speeds produce an overabundance of friction heat, which decreases the composite’s mechanical strength and load-supporting capacity and, subsequently, its wear resistance.

### 3.4. Effect of Applied Load on Wear and Coefficient of Friction

**Figure 6:**
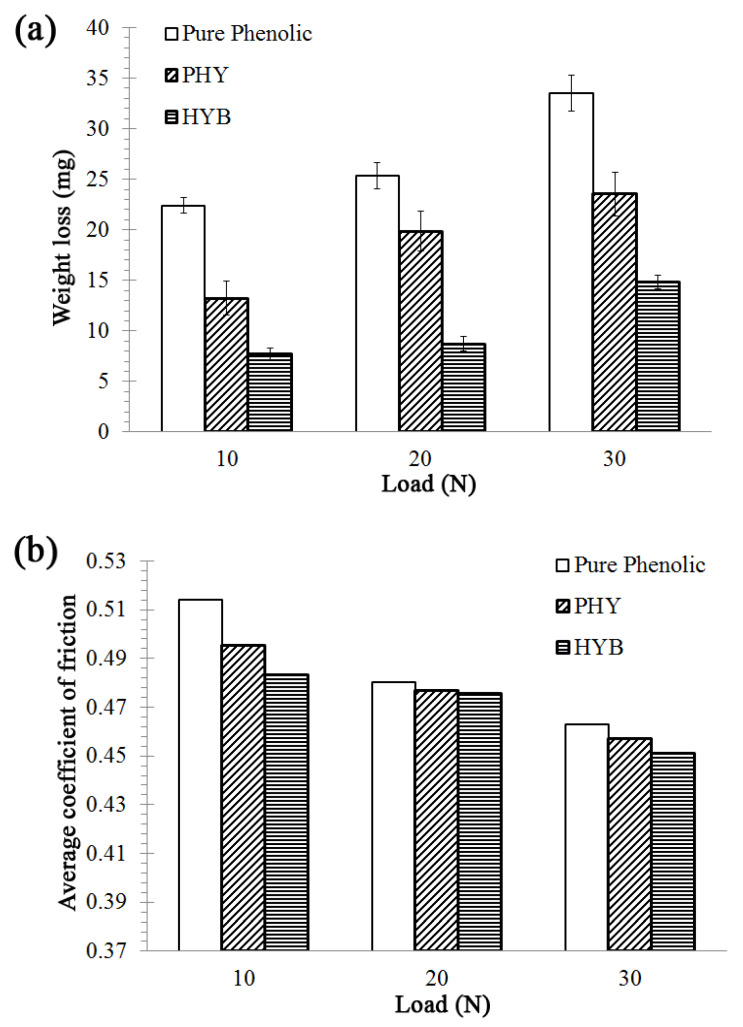
Properties of the HYB composite and PHY composite after wear test for different materials with the variation of applied load (N) (Sliding speed: 1.022 m/s, Filler: 5 wt%): (**a**) weight loss and (**b**) average coefficient of friction.

The effect of applied loads on the wear behaviour and average COF of pure phenolic and on the HYB composite and PHY composite with 5 wt% filler at the sliding speed of 1.022 m/s under three different loads (10, 20 and 30 N) was also investigated, and the results are shown in Figure 6. In the case of all phenolic composites, the wear (weight loss) increases with increasing applied load (Figure 6), whereas the average COF decreases with increasing applied load (Figure 6). This is because, at higher applied loads, the real contact area between the composite and abrasive surfaces increases. Therefore, increasing the applied load increases the penetration depth of the abrasive asperities to the composite contact surface, increasing the real contact area and the wear (weight loss) of all composites.

The HYB composite showed a minimum weight loss of 7.71 mg, 8.72 mg, and 14.85 mg, respectively. The average COF was reduced by ~9.94% from 10 to 30 N applied loads for pure phenolic, ~7.68% in the PHY composite, and ~6.64% in the HYB composite. The weight loss and the average COF of the composites are in the order of HYB composite < PHY composite < pure phenolic under an applied load. Thus, it can be surmised that the HYB composite influenced the improvement of wear resistance. It was proposed that the CNTs/alumina, well-distributed in the HYB composite, acted as a lubricant for wear reduction and supported the bonding between the filler and matrix, thus improving the load-carrying capacity of the HYB composite. The composite surface temperature increases quickly when under a high applied load, which softens the composite surface. Surface softening is caused by the frictional-induced heat at the sliding interface and the reduction in the average COF due to a smearing effect. Hence, the average COF decreases when the applied load increases [49].

### 3.5. FESEM of Worn Surfaces of the Composite

**Figure 7:**
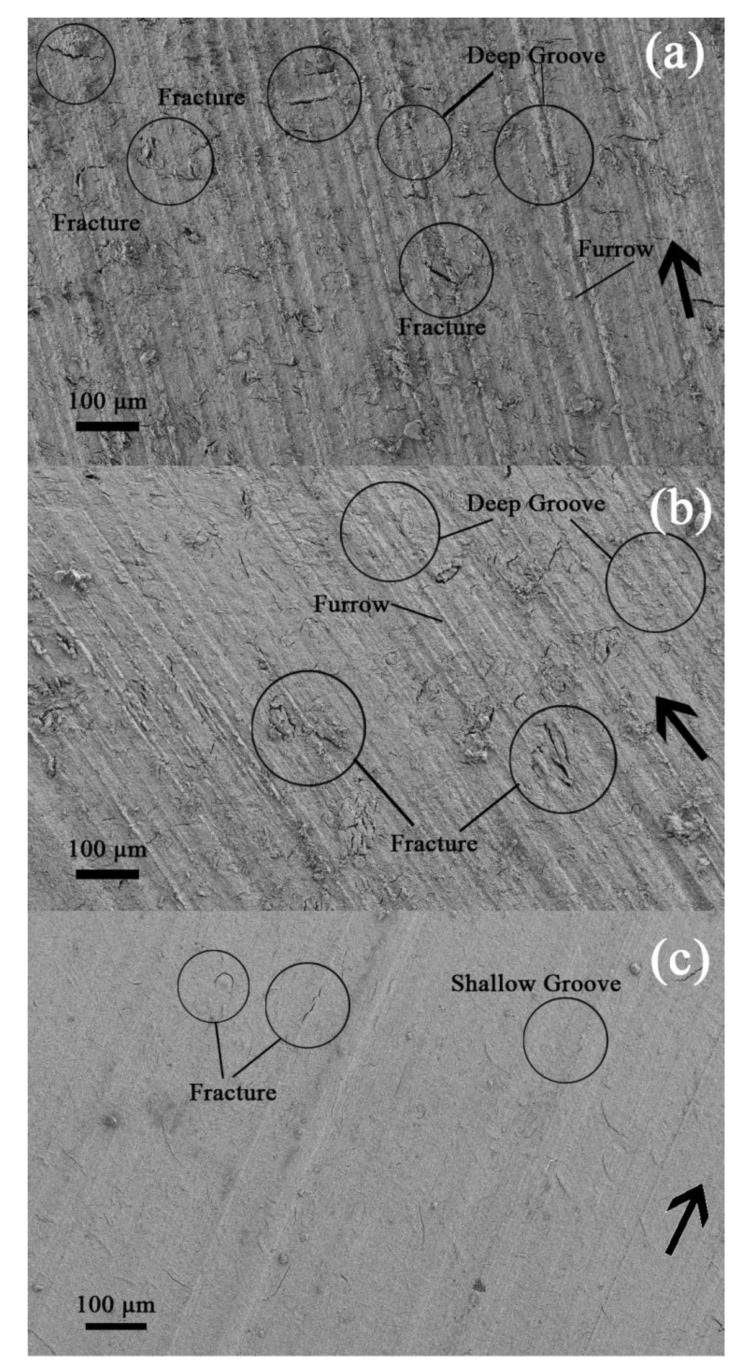
SEM micrographs of the worn surfaces for HYB composite and PHY composite under a sliding speed of 1.022 m/s and 30 N applied load. (**a**) Pure phenolic, (**b**) 5 wt% PHY composite, (**c**) 5 wt% HYB composite. → sliding direction.

Figure 7a–c shows the FESEM micrographs of the pure phenolic (worn surfaces), PHY composite, and HYB composite under 30 N applied load and a sliding speed of 1.022 m/s. The arrows represent the sliding directions. The penetration of SiC hard asperities from the abrasive paper into the sample’s surface during the sliding process would produce grooving wear, ploughing, plastic deformation, and surface fracture. Figure 7a shows the furrows, deep groove, and fracture on the pure phenolic worn surface. The surface shows plastic flow caused by micro-cutting and micro-ploughing processes during sliding. Therefore, it can be surmised that abrasive and adhesive wear are the primary mechanisms in the phenolic under abrasive conditions. By adding the CVD hybrid filler, the amount of damage on the worn surface of the phenolic composite was minimised, which improved the wear resistance, per Figure 7c, which shows that the worn surface of the HYB composite is smoother than the PHY composite. In addition, the grooves on the worn surface appear shallower, reducing the fracture. This shows that the wear mechanism transforms to mild abrasive and mild adhesive wear after reinforcement with CVD hybrid compound with phenolic. This is due to better stress transfer between filler and matrices. The CVD hybrid would act as a stress transfer medium to resist removing composite surface materials from the bulk composite body.

### 3.6. Hardness of the Composite

**Figure 8:**
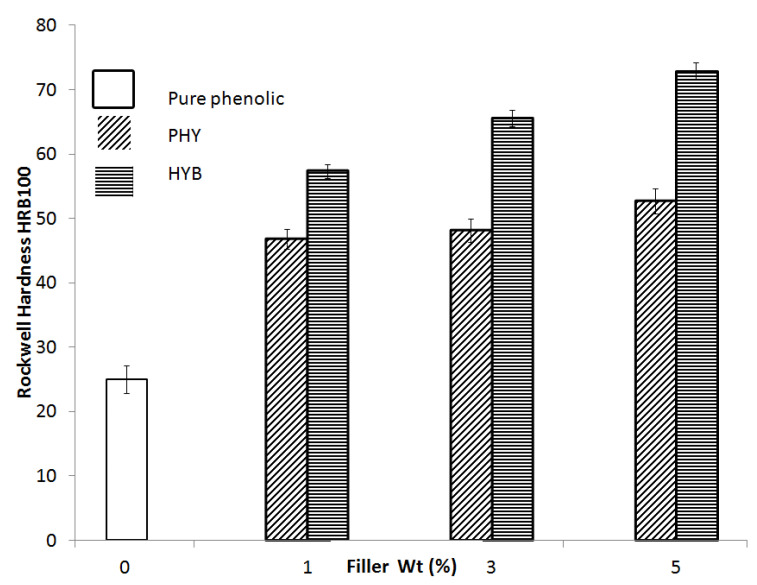
Hardness number of the HYB composite and PHY composite as a function of filler loading.

Hardness is a parameter expressing the deformation resistance of a material against a concentrated force on its surface. Figure 8 shows the micro-hardness number of pure phenolic, HYB composites, and PHY composites as a function of filler loading. With increasing filler loadings, the hardness value increases progressively for both HYB and PHY composites. The improvement of the hardness number can be attributed to the CNTs and alumina fillers in the composite. In addition, the distance between the filler particles will decrease as the loadings increase and restrict plastic deformation. During the test, the micro-hardness value of the HYB composites was higher than that of the PHY composite. The HYB composite micro-hardness value for all samples is higher at 5 wt% filler loading, with an increment of 192.1%. Meanwhile, the PHY composite micro-hardness value at 5 wt% filler loading reported an increment of 111.26%. This improvement can be attributed to the hybridisation of carbon nanotubes (CNTs) with alumina microparticles. Furthermore, the higher stiffness and rigidity of the MWCNTs improved the hardness number of the HYB composite significantly. Generally, surface hardness is one of the most important aspects that govern material wear resistance. A harder surface has a higher wear resistance. The highest filler loading for the CVD hybrid filler reported the highest hardness and wear resistance.

## 4. Conclusions

The wear resistance and hardness of the phenolic composite were improved with the addition of the hybrid compound produced by both CVD and physical methods. Also, phenolic composites’ tribological properties and hardness were further enhanced by hybridising the CNTs with alumina via CVD (CVD hybrid) compared to the physical hybrid. HYB composites showed better wear resistance due to the low volume and average COF compared to the PHY composites. The HYB hybrid compound dispersed well in the matrix, thus improving its tribological properties. 5 wt% filler loading for HYB composites showed the highest wear resistance and the lowest average COF among the samples. The HYB composite showed a minimum weight loss of 4.68 mg and a 74.68% reduction of wear (weight loss) compared to pure phenolic. The worn surface of the 5 wt% filler-loading HYB composite was smooth, indicating that the structure of the phenolic filled with HYB is not easily detached when sliding against an adhesive surface. The hardness of the HYB composite was higher than that of the PHY composites. Compared with pure phenolic, the hardness of the HYB composite with 5 wt% filler loading increased by 192.10%, whereas that of the PHY composite increased by 111.26% due to the combined effect of the hybrid filler produced via the CVD method.

## Figures and Tables

**Table 1:** Weight percentages of carbon, oxygen, nickel and aluminium of CNTs/alumina hybrid synthesis using the CVD method.

Element	Weight Percentage (wt%)
Carbon	23.12
Oxygen	25.54
Nickel	3.84
Aluminium	47.5

**Table 2:** Descriptions for sample HYB and PHY (1–5 wt% filler loading).

Samples	Fillers
HYB	Phenolic filled with CNTs/Alumina CVD hybrid compound (produced via CVD method)
PHY	Phenolic filled with CNTs physically hybrid compound (produced via the physical milling method)

## Data Availability

Not applicable.
